# Safety and Efficacy Profiles of the Live Attenuated Vaccine AVAC ASF LIVE for Preventing African Swine Fever in Pigs

**DOI:** 10.1155/tbed/8623876

**Published:** 2025-06-20

**Authors:** Nguyen Van Diep, Nguyen Thi Ngoc, Nguyen Van Duc, Vu Xuan Dang, Tran Ngoc Tiep, Chu Thi Quy, Bui Thi Tham, Pham Ngoc Doanh

**Affiliations:** ^1^Center for Research and Development of Veterinary Vaccines, AVAC Viet Nam Joint Stock Company, Hung Yen, Vietnam; ^2^Institute of Biology, Vietnam Academy of Science and Technology, Hanoi, Vietnam

**Keywords:** African swine fever, efficacy, safety, vaccine

## Abstract

African swine fever (ASF) is one of the most devastating diseases affecting the global pig industry. Therefore, the development of safe and effective vaccines is crucial in combating the virus. The AVAC ASF LIVE vaccine, produced from an attenuated genotype II ASF virus (ASFV) strain with the deletion of six MGF genes and cultured in a Diep's macrophage (DMAC) cell line, has been officially licensed for use and commercialization in Vietnam. This study evaluated the safety and efficacy of the AVAC ASF LIVE vaccine. In the safety experiment, pigs received a dose equivalent to 100 times the protective dose. In the efficacy experiment, control pigs and one-dose vaccinated pigs were challenged with a highly virulent p72 genotype II ASFV on day 28 post-vaccination. The duration of protective immunity was assessed by challenging pigs at various time points, from 2 weeks up to 6 months post-vaccination. Results showed that pigs given the 100-fold protective dose remained healthy with no abnormal signs. Significantly, 60% of vaccinated pigs survived the challenge 14 days after vaccination, and the survival rates reached 100% when challenged at 28, 90, 120, and 150 days post-vaccination (dpv). The vaccine effectively induced robust immunity, leading to a reduction in viral shedding and the persistence of viral DNA in vaccinated animals. In conclusion, the AVAC ASF LIVE vaccine has demonstrated safety and high efficacy in protecting pigs from genotype II ASFV infection.

## 1. Introduction

African swine fever (ASF) is a transboundary infectious swine disease caused by the ASF virus (ASFV) of the genus *Asfivirus*. The virus has a large linear double-stranded DNA genome ranging from approximately 170–190 kbp [1]. ASFV has been classified into 24 genotypes based on sequence variation in the C-terminal region of the B646L gene, which encodes the major capsid protein p72. Among these, genotype II ASFV is the most prevalent, exhibiting various levels of virulence, but is generally considered highly virulent and responsible for global ASF outbreaks [2–4]. The disease affects pigs of all breeds and ages, manifesting in peracute, acute, subacute, or chronic conditions. The acute disease is characterized by a sudden onset of high fever, loss of appetite, purplish skin, severe internal hemorrhage, and a high mortality rate of up to 100% [5].

The first documented description of ASF was in Kenya in 1921 [6]. However, a retrospective study suggested that the first outbreak actually occurred in 1907. Initially, the disease was limited to Africa until 1957, when it spread to Portugal, marking its introduction into Europe. Over time, ASF rapidly spread to other European countries. By 2007, almost all countries had successfully eradicated ASF except for Italy and Africa. However, in 2007, a strain of genotype II ASFV emerged in the Republic of Georgia and quickly spread to other Transcaucasian countries, triggering worldwide outbreaks. As of 2024, ASF remains widespread in different regions of Africa, the Americas, Asia, and Europe, affecting both domestic and wild pig populations [7]. Global outbreaks have had a devastating impact on swine production, leading to significant economic losses for the industry [7–9]. Consequently, the primary priority for the pig industry is the control of ASF.

In the case of infectious diseases like ASF, the most effective strategy for control is a combination of strict biosecurity measures and vaccination. However, controlling ASF has primarily relied on biosecurity measures. Unfortunately, ASFV can be transmitted through various routes, including direct and indirect transmission [9]. Wild boars and ticks serve as natural reservoirs of ASFV, contributing to its spread through the forest cycle. The virus can spread between wild and domestic pigs, leading to increased infections in both populations. In the domestic cycle, domestic pigs are the primary hosts and carriers of the virus. Other animals like flies, leeches, and birds can also act as carriers of the virus over long distances. Human activities, such as illegal transportation of infected pork products and contaminated items, contribute to the spread of ASFV. Additionally, ASFV is highly resistant to environmental conditions, allowing it to persist and spread over long distances. These factors complicate ASF prevention and control efforts [9]. The rapid and worldwide spread of ASF indicates that current methods, primarily based on biosecurity measures, are not sufficient and highlights the urgent need for effective vaccines to combat ASF [7]. In fact, numerous efforts have been made to develop vaccine candidates against ASF [9]. However, inactivated vaccines have been proven to be ineffective against ASF [9, 10]. Developing genetically engineered vaccines, such as subunit vaccines, vector vaccines, and DNA vaccines, has faced enormous challenges due to the complexity of ASFV. Most experiments with these vaccines have focused on assessing immunogenicity and have not included challenge experiments [10]. Despite concerns about the safety and instability of live attenuated vaccines, many candidates are being studied [9]. Experimental results indicated that both humoral and cell-mediated immunities play a role in protection [10]. However, reports suggest that the late onset and short duration of protection induced by live attenuated vaccines, along with safety concerns, have hindered their progression beyond the laboratory research stage [9, 10]. Recently, two live-attenuated vaccines, AVAC ASF LIVE (AVAC Viet Nam JSC, Hung Yen, Vietnam) and NAVET-ASFVAC (Navetco National Veterinary JSC, Ho Chi Minh, Vietnam), have been licensed for nationwide use in Vietnam since 2023 [11, 12]. Here, we present the safety and efficacy data for the AVAC ASF LIVE vaccine.

## 2. Materials and Methods

### 2.1. Materials

#### 2.1.1. Vaccine Virus

The vaccine virus was developed by further attenuating the ASFV-G-ΔMGF strain in Diep's macrophage (DMAC) cell line. ASFV-G-ΔMGF is an attenuated strain derived from the highly virulent ASFV Georgia 2007 isolate (ASFV-G) by deleting six genes (MGF505-1R, MGF360-12L, MGF360-13L, MGF360-14L, MGF505-2R, and MGF505-3R) [13]. The DMAC cell line, developed by AVAC VIETNAM JSC, is a natural cell line selected from macrophages of pig fetuses for high sensitivity to ASFV [12]. We obtained the attenuated ASFV-G-ΔMGF strain from the United States Department of Agriculture under license number 78/TY-QLT, issued by the Department of Animal Health of Vietnam on January 29, 2021. We further attenuated ASFV-G-ΔMGF by propagating it to 30 successive passages in DMAC cells to create the master virus seed for the vaccine, designated as ASFV-G-ΔMGF-DMAC. The genome sequence of ASFV-G-ΔMGF-DMAC was deposited in the GenBank database under accession number PP529961. A single dose of the AVAC ASF LIVE vaccine contains approximately 10^4^ HAD_50_ ASFV-G-ΔMGF-DMAC.

#### 2.1.2. Challenge Virus

The highly virulent ASFV (Hv-Avac01) used in the challenge experiments was isolated from a pig that died during an ASF outbreak in Hung Yen, Vietnam, in 2019. The pig showed typical symptoms and pathology of acute ASF, including high fever, sudden loss of appetite, anorexia, lethargy, respiratory distress, disseminated cyanosis, and an enlarged spleen. The Hv-Avac01 strain is classified as p72 genotype II ASFV, and its genome sequence is deposited in GenBank under accession number PV339937.

#### 2.1.3. Animals

Healthy, 4-week-old, crossbred (Yorkshire–Landrace–Duroc) pigs were purchased from a high-quality commercial breeding farm in Bac Giang province for the experiments. Upon arrival, each pig was ear-tagged. All pigs underwent testing to confirm they were negative for ASF, porcine circovirus-2, foot-and-mouth disease, classical swine fever, and porcine reproductive and respiratory syndrome viruses using quantitative polymerase chain reactions (qPCR). Additionally, the pigs were tested to confirm they were negative for anti-ASFV antibodies using enzyme-linked immunosorbent assay (ELISA). They were given a 3-day acclimatization period before the start of the experiments. The rectal temperature of each pig was measured for three consecutive days prior to each experiment to calculate the mean baseline temperature for the group. The pigs were randomly assigned to groups and housed in appropriate containment with optimal animal welfare conditions at the Avac company's animal facility. They were provided with age-appropriate commercial feed and water ad libitum and monitored daily.

### 2.2. Experimental Designs

In this study, we conducted three experiments to assess the safety of the vaccine in young animals when administered in an overdose, the protective efficacy of the vaccine, and the duration of protective immunity. The experimental designs were consistent with the WOAH [14] draft guidelines.

#### 2.2.1. Evaluating the Safety of the Vaccine in Young Animals When Administered in an Overdose

The safety evaluation of AVAC ASF LIVE was conducted in 20 young pigs aged 4 weeks. Ten pigs (ear-tag: S1–S10) were intramuscularly vaccinated with a dose of 10^6^ HAD_50_ ASFV-G-ΔMGF-DMAC in 2 mL of diluent per pig (equivalent to 100 doses of the protective dose). The control group comprised 10 pigs (ear-tag: S11–S20) intramuscularly inoculated with 2 mL of sterile phosphate-buffered saline (PBS) per pig. The pigs were monitored daily for 45 days post-vaccination (dpv) for clinical signs, including anorexia, recumbence, purple skin discoloration, joint swelling, ocular discharge, labored breathing, coughing, and digestive findings [12]. Additionally, the rectal body temperature of each pig was measured daily in the morning using a digital thermometer while the pigs were eating. Fever was defined as a rectal temperature above the baseline by 1.5°C [14], which was determined to be 40.5°C in this study. Daily clinical scores for each animal were recorded using a clinical scoring system, which was calculated by summing the scores of eight clinical signs: fever, anorexia, recumbence, skin hemorrhage, joint swelling, labored breathing and/or coughing, ocular discharge, and digestive findings [12, 15, 16]. Each clinical sign was categorized into four levels: normal, mild, moderate, and severe, with corresponding scores of 0, 1, 2, and 3, respectively. Pigs that showed persistent fever (>40.5°C), severe anorexia, recumbency, respiratory distress, or neurological symptoms for more than two consecutive days, or reached a clinical score of >18, were humanely euthanized by intravenous pentobarbital injection for examination [15, 16]. At the end of the experiments or after reaching the humane endpoints, all pigs were euthanized by intravascular injection of Pentobarbital following deep anesthesia and subjected to postmortem examination. Gross findings focused on the spleen, lung, kidney, tonsil, intestinal tract, and mesenteric, gastrohepatic, and submandibular lymph nodes. The experimental protocol was summarized in Table 1.

#### 2.2.2. Evaluating the Efficacy of the Vaccine

An experiment was conducted to assess the efficacy of the vaccine using 15 pigs. Ten pigs (ear-tag: E1–E10) received an intramuscular vaccination with a vaccine dose of 10^4^ HAD_50_ ASFV-G-ΔMGF-DMAC in 2 mL of diluent per pig, while the remaining five pigs (ear-tag: E11–E15) received an intramuscular injection of 2 mL of sterile PBS per pig as a control group. All pigs were monitored daily for clinical signs and rectal temperatures for a 28-day period. Whole blood and serum samples were collected at 7, 14, 21, and 28 dpv to test for viremia and anti-ASFV antibodies, respectively. Additionally, oral–nasal and rectal swabs were collected at these time points to test for viral shedding through secretions.

After the sample collection on day 28 post-vaccination, all pigs were intramuscularly challenged with virulent ASFV (Hv-Avac01) at a dose of 10^3^ HAD_50_ per pig. The pigs were then observed for 45 days post-challenge (dpc) to detect any clinical signs of acute or chronic disease using the clinical scoring system. Whole blood, serum, oral–nasal, and rectal swab samples were collected at 4, 7, 10, 14, 21, 28, 35, and 45 dpc to test for ASFV and anti-ASFV antibodies (for serum sample). Any pigs that reached the humane endpoint were intravenously euthanized using 10 mL of Pentobarbital (240 mg/mL) following deep anesthesia. Euthanized pigs and any pigs that died underwent a thorough necropsy to determine the cause of death, with tissues collected for ASFV detection. After 45 days of monitoring, all surviving experimental pigs were humanely euthanized and necropsied to examine gross pathology. Tissue samples, including spleen, kidney, lung, and submandibular lymph node, were collected to detect ASFV persistence. The experimental protocol was summarized in Table 2.

#### 2.2.3. Determination of Duration of Protective Immunity

An experiment was conducted to determine the duration of protective immunity involving 46 healthy 4-week-old pigs. They were divided into two groups: the vaccinated group consisted of 28 pigs (ear-tag: D1–D28), each of which received an intramuscular injection of one vaccine dose containing 10^4^ HAD_50_ of ASFV-G-ΔMGF-DMAC. The control group included 18 pigs (ear-tag: D29–D46), each of which was injected intramuscularly with 2 mL of sterile PBS. Both groups of pigs were raised in separate pens under identical conditions. Serum samples were collected at 14, 28, 90, 120, 150, and 180 dpv to test for anti-ASFV antibodies. At each time point of 14, 90, 120, 150, and 180 dpv, five pigs from the vaccinated group and three from the control group were randomly selected to be challenged with virulent ASFV (Hv-Avac01) via intramuscular injection at a dose of 10^3^ HAD_50_ per pig. The challenged pigs were monitored daily for a period of 21 days for clinical signs. If a pig died or reached the humane endpoint, a necropsy was conducted to examine gross lesions and determine the cause of death. Pigs were considered protected by the vaccine if they survived the challenge in good health, showing no typical clinical signs of acute or chronic ASF throughout the observation period. The survival rate of pigs post-challenge was calculated. The experimental protocol was summarized in Table 3.

### 2.3. Methods

#### 2.3.1. Analysis of the ASFV-G-MGF-DMAC Genome Sequence

Genome sequence of the ASFV-G-MGF-DMAC virus was analyzed using next-generation sequencing (NGS) technology [12]. DNA extraction was performed using the QIAamp DNA Blood Mini Kit (QIAGEN, Hilden, Germany). The DNA library was prepared with the TruSeq DNA Nano 350bp library Kit (Illumina, California, USA) and sequenced on the Illumina Novaseq600 instrument (Illumina, California, USA). The raw sequencing data were processed using Fastp v0.23.4 [17] to remove low-quality reads with a threshold of 50 nt. Host-derived reads were filtered out by aligning them with the pig genome (GenBank accession no. GCF_000003025.5) using Bowtie2 v2.5.3 [18] and Samtools v1.15.1 [19]. The reads, after the removal of host-derived material, were de novo assembled with Unicycler v0.5.0 [20], and the quality of the assembly was assessed using CheckV v1.0.3 [21]. The reads were mapped back to the genome to determine the coverage. The assembled genome was annotated using Prokka v1.14.6 [22].

#### 2.3.2. Collection of Oral–Nasal and Rectal Swabs

Oral–nasal and rectal swabs were collected at indicated time points to test for viral shedding through secretions using dry transport swabs (code: HP5005, Jiangsu Huida Medical Instruments Co., Ltd - Jiangsu, China).

#### 2.3.3. Antibody Detection

Serum samples were tested for anti-ASFV antibodies by ELISA using the VDPro ASFV Ab i-ELISA Version 2.0 Kit (MEDIAN Diagnostics, Seoul, Korea) following the manufacturer's instructions. Results were considered positive if *S*/*P* ≥ 0.25 and negative if *S*/*P* < 0.25.

#### 2.3.4. Virus Detection Before Experiments

All pigs were tested to confirm they were negative for ASF, porcine circovirus-2, foot-and-mouth disease, classical swine fever, and porcine reproductive and respiratory syndrome viruses before experiments. Viral nucleic acids were extracted from blood samples of pigs using the Virus DNA/RNA Extraction Kit 2.0 (Vazyme, China) following the manufacturer's instructions. The detection of the specified viruses was performed using qPCR with corresponding commercial kits: VDx ASFV qPCR Ver 2.1, VDx PCV2 qPCR, VDx FMDV 3Diff/PAN qRT-PCR set_FMDV qRT-PCR premix (A), VDx CSFV qRT-PCR, and VDx HP-PRRSV qRT-PCR Ver 1.1 (MEDIAN Diagnostics Inc., Seoul, Korea).

#### 2.3.5. ASFV Detection and Differentiating Vaccine Virus and Challenge Virus in Experimental Pigs

Blood, swab, and tissue samples of experimental pigs were tested for ASFV at specified time points according to the experimental design. Viral nucleic acids were extracted from the samples using the Virus DNA/RNA Extraction Kit 2.0 (Vazyme, China) following the manufacturer's instructions. ASFV detection was performed by amplifying the B646L gene in a qPCR using the commercial Realtime PCR Kit VDx ASFV qPCR ver 2.0 (MEDIAN Diagnostics, Seoul, Korea). A cycle threshold (Ct) value below 38 was considered positive.

In cases where the B646L test was positive, two additional separate qPCRs were carried out to identify the specific ASFV strain by detecting the presence or absence of the MGF360-12L and beta-glucuronidase (β-GUS) genes. The ASFV field strain is characterized by the presence of the MGF360-12L gene, while the vaccine virus is distinguished by the presence of the β-GUS gene. The primers and probes for detecting these genes were previously described [23] as follows:

For MGF360-12L detection: primers (forward, MGF360-12LF 5′- CATACCCTTCCCCTAAAGCTG-3′; reverse, MGF360–12LR 5′-CTACTGCTATGTCCTGGGC-3′), and MGF360–12L probe (5′-FAM-ACCCTCTTCGAAAACATCAGCCCC-BHQ1-3). For β-GUS detection: primers (forward, 5′-TCTACTTTACTGGCTTTGGTCG-3′; reverse, 5′-CGTAAGGGTAATGCGAGGTAC-3′), and GUS probe (5′-FAM-AGGATTCGATAACGTGCTGATGGTGC-BHQ1-3′).

Each qPCR reaction included 1 μL of DNA template, 0.4 μM of each primer, 0.2 μM of the probe, and 10 μL GoTaq Probe qPCR Master Mix (Promega). The reaction volume was adjusted to 20 μL with nuclease-free water. The thermal cycling protocol included an initial denaturation step at 95°C for 2 min, followed by 40 cycles of 95°C for 15 s and 60°C for 1 min. All reactions were performed on a Bio-Rad CFX Opus 96 Real-Time PCR System. A sample was considered positive if the Ct value was below 38; samples with no Ct value were recorded as negative and marked as “−”.

### 2.4. Statistical Analysis

Statistical analyses, when applicable, were performed using SPSS Statistics. The *t*-test was utilized to evaluate group differences. A *p*-value of < 0.05 was considered statistically significant.

## 3. Result

### 3.1. Genome Sequence of ASFV-G-ΔMGF-DMAC and Compared to the ASFV-G-ΔMGF

To accurately assess the changes in the ASFV-G-ΔMGF-DMAC virus compared to the original ASFV-G-ΔMGF strain, the genome sequence was analyzed using NGS technology and compared with the published ASFV-G-ΔMGF strain in GenBank. The sequencing results revealed that the complete genome of ASFV-G-ΔMGF-DMAC consists of 184,187 nucleotides with an average coverage of 1049x and a G + C content of 38.60%. The sequence has been deposited in GenBank under the accession number PP529961. Comparative analysis of the gene sequence with the ASFV-G-ΔMGF (GenBank accession number KH481870) showed a 99.90% similarity. No changes were observed in the gene identity region: deletion of 6 genes from the multigene family (MGF360 and MGF505) and the insertion of the P72 β-GUS marker. However, specific mutations were identified in various gene regions, resulting in frame shifts, amino acid changes, and stop codons. Furthermore, 51 single-nucleotide polymorphisms (SNPs) were detected in nonprotein-coding regions. The detailed mutations and their effects are summarized in Table 4.

### 3.2. The Safety of the AVAC ASF LIVE Vaccine in Young Animals

**Figure 1:**
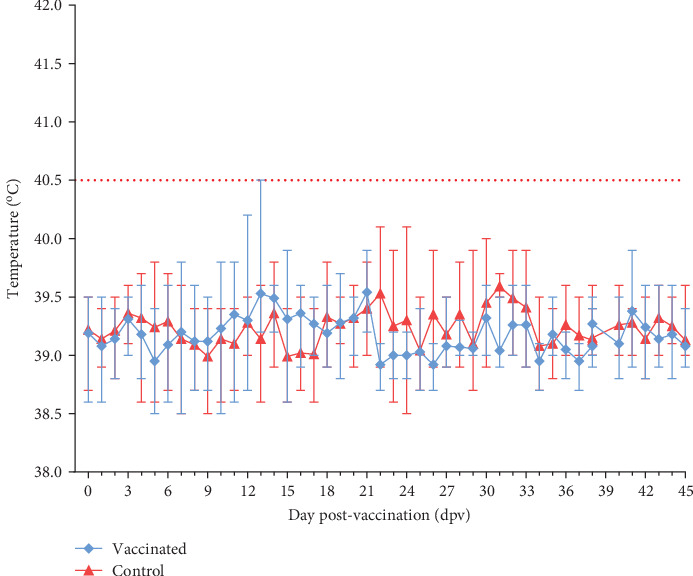
Daily rectal temperature of pigs during 45 days post-vaccination. Above 40.5°C is considered fever.

**Figure 2:**
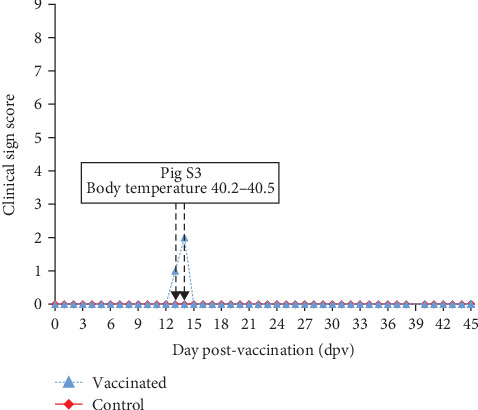
Daily average clinical scores of pigs during 45 days post-vaccination.

The safety test of the AVAC ASF LIVE vaccine in young animals after an overdose vaccination equivalent to 100 times the protective dose showed that all vaccinated pigs remained healthy during the 45-day observation period. Their body temperatures were within the normal range, similar to control pigs (*p* > 0.05), as shown in Figure 1. They had good appetites, normal activities, and clinical scores of zero, similar to the control group. The only exception was pig S3, which exhibited a slight increase in body temperature for 2 days, resulting in a low score (below 2.0) as shown in Figure 2. Necropsy findings on day 45 post-vaccination revealed no abnormal pathological changes in any of the vaccinated pigs.

### 3.3. The Efficacy of the AVAC ASF LIVE Vaccine

In the efficacy test, 10 pigs (ear-tagged E1–E10) received a vaccine dose, while five pigs (ear-tagged E11–E15) served as the control group. All pigs were monitored daily for clinical signs over a 28-day period and tested for viremia and anti-ASFV antibodies at 7, 14, 21, and 28 dpv. After sample collection on day 28 post-vaccination, all pigs were challenged with virulent ASFV. The pigs were observed for 45 dpc for any signs of acute or chronic disease and tested for ASFV and anti-ASFV antibodies. Any pigs that died or were euthanized after reaching the humane endpoint underwent necropsy to determine the cause of death, with tissues collected for detection of ASFV persistence. The same procedure was followed for all surviving experimental pigs after 45 days of monitoring. The results are as follows:

#### 3.3.1. Clinical Signs Post-Vaccination and Post-Challenge, and Survival Rate Post-Challenge

**Figure 3:**
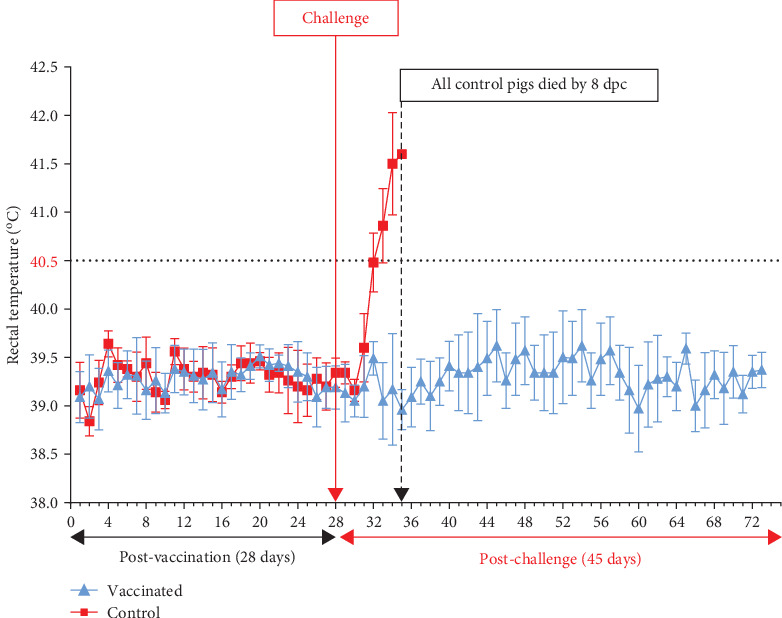
Daily rectal temperature of pigs post-vaccination and post-challenge.

**Figure 4:**
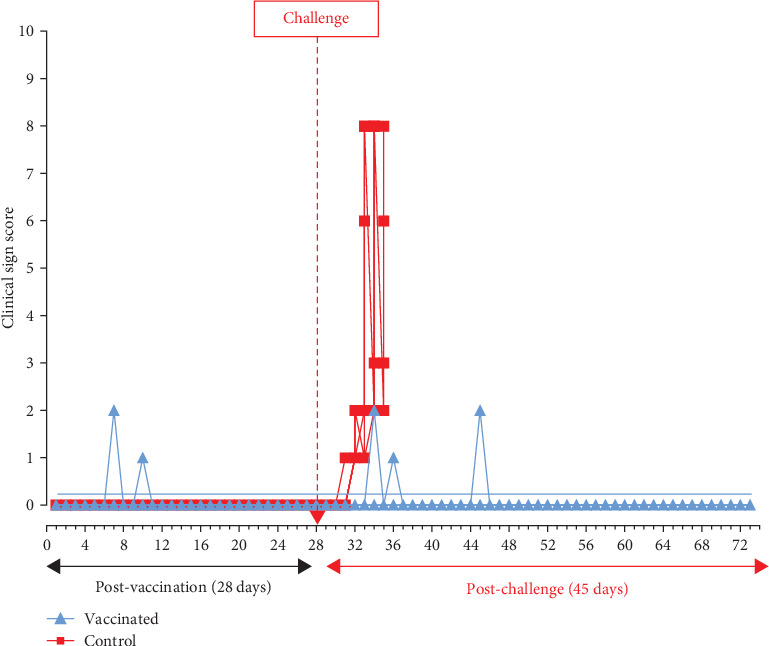
Daily average clinical scores of pigs post-vaccination and post-challenge.

**Figure 5:**
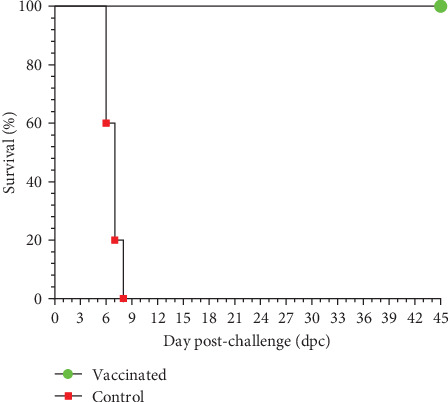
Survival rate of pigs post-challenge.

During the 28-day observation period following vaccination, all vaccinated pigs exhibited normal activities and had rectal temperatures comparable to the control group (*p* > 0.05). None of the vaccinated or control pigs experienced a fever prior to the challenge, as depicted in Figure 3, resulting in zero or very low clinical scores (Figure 4). However, the health status of vaccinated pigs and control pigs differed significantly post-challenge. All vaccinated pigs remained healthy, showing no abnormal clinical manifestations, except for two pigs (E4 and E5) that had a poor appetite for 1–2 days on 7 and 9 dpc. Afterward, they returned to normal activities, achieving a 100% survival rate (Figure 5). The necropsy results at 45 dpc did not reveal any lesions in the internal organs of the vaccinated pigs. In contrast, starting from day 3 post-challenge, control pigs experienced fever (>40.5°C) (Figure 3). Subsequently, the disease symptoms worsened, including high fever, loss of appetite, reduced movement, and diarrhea, resulting in high clinical scores (Figure 4). There were significant differences in body temperature and clinical scores between vaccinated and control pigs (*p*-values < 0.05 in both datasets). All five control pigs died from 6 to 8 dpc (Figure 5). Necropsy examination revealed typical ASF lesions, such as splenomegaly, swollen hemorrhagic lymph nodes, petechiae in the renal cortex, and hemorrhagic lungs.

#### 3.3.2. Viremia Post-Vaccination and Post-Challenge

**Figure 6:**
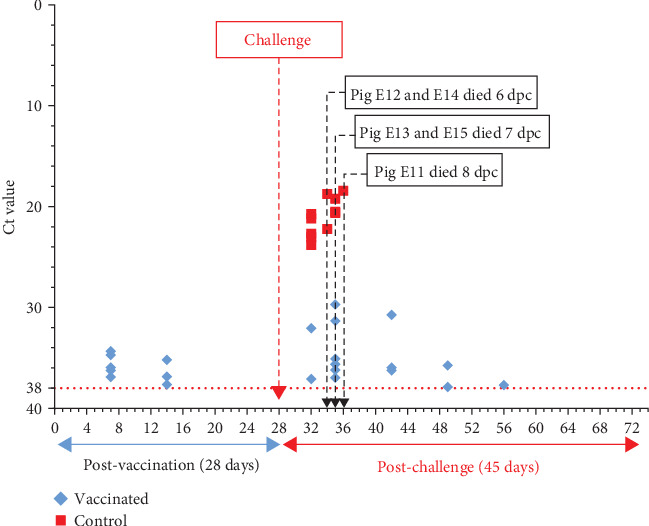
Viremia was detected in pigs post-vaccination and post-challenge.

The analysis of blood samples from vaccinated and control pigs at 7, 14, 21, and 28 dpv revealed that five out of 10 vaccinated pigs developed viremia at 7 dpv, with 3 pigs remaining viremic at 14 dpv (Figure 6). ASFV DNA in these positive samples was low, indicated by very high Ct values ranging from 34.32 to 37.29. By 21 dpv, all blood samples from vaccinated pigs were negative for ASFV. In contrast, all blood samples from the control group were negative for ASFV at all time points (Figure 6).

Following the challenge, all control pigs exhibited viremia starting from the first sampling point at 4 dpc, with low Ct values ranging from 20.71 to 23.80, indicating high ASFV DNA. Ct values decreased further as pigs succumbed to the infection, with the lowest Ct value recorded at 18.42 (Figure 6). Among vaccinated pigs, ASFV was detected in 2 out of 10 (20.0%) pigs at 4 dpc, increasing to 6 (60.0%) pigs at 7 dpc, and then decreasing to 4 (40.0%) pigs at 14 dpc, 2 (20.0%) pigs at 21 dpc, and 1 (10.0%) pig at 28 dpc. Ct values of the positive samples were high, ranging from 29.70 to 37.68, indicating low ASFV DNA. All blood samples collected from vaccinated pigs at 35 and 45 dpc tested negative for ASFV (Figure 6).

#### 3.3.3. Viral Shedding in Secretions Post-Vaccination and Post-Challenge

**Figure 7:**
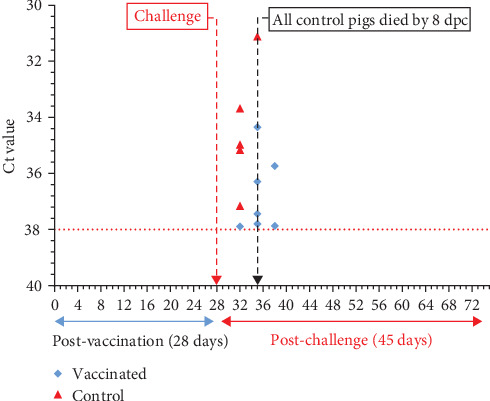
ASFV detection in oral–nasal samples of pigs post-vaccination and post-challenge.

After vaccination, all oral–nasal swab and rectal swab samples from both vaccinated and control pigs were negative for ASFV at all sampling time points of 7, 14, 21, and 28 dpv (Figure 7). Following the challenge, ASFV DNA was detected in all oral–nasal swab samples from control pigs until their death, with Ct values ranging from 28.69 to 34.17 (Figure 7). In vaccinated pigs, ASFV DNA was detected in 1 out of 10 (10.0%) pigs at 4 dpc, in 4 (40.0%) pigs at 7 dpc, and in 2 (20%) pigs at 10 dpc, with high Ct values ranging from 35.35 to 37.89. However, all pigs tested negative for ASFV at later time points of 14, 28, 35, and 45 dpc (Figure 7). All rectal swab samples from vaccinated pigs were negative for ASFV post-challenge.

#### 3.3.4. Persistence of ASFV in Internal Organs Post-Challenge

On day 45 post-challenge, vaccinated pigs were necropsied, and internal organs (spleen, kidney, lung, and lymph nodes) were collected to detect the vaccine and challenge ASFV DNA by qPCR. The results indicated that ASFV DNA was present in the organs of four pigs (E2, E4, E5, and E7), with Ct values ranging from 32.0 to 36.8 based on the B646L gene (Table 5). Among these, samples from pigs E4 and E7 tested positive for the MGF360-12L gene and negative for the β-GUS gene, confirming the presence of only challenge ASFV DNA. Meanwhile, pig E5 tested negative for the MGF360-12L gene and positive for the β-GUS gene, indicating the presence of only vaccine ASFV DNA. In the case of pig E2, although three samples (spleen, kidney, and lymph node) were positive for the B646L gene (Ct values above 36.0), they were negative for both the MGF360-12L and β-GUS genes. Therefore, the identification of the vaccine or challenge virus in the samples of pig E2 was inconclusive.

#### 3.3.5. Anti-ASFV Antibodies

**Figure 8:**
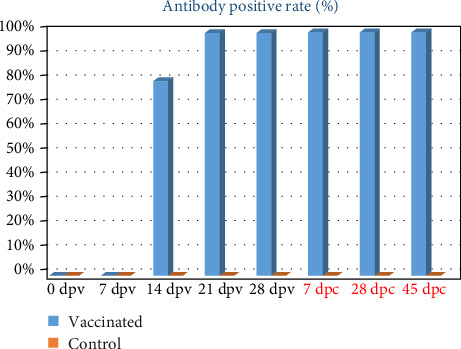
Positive rate for anti-ASFV antibodies post-vaccination and post-challenge.

**Figure 9:**
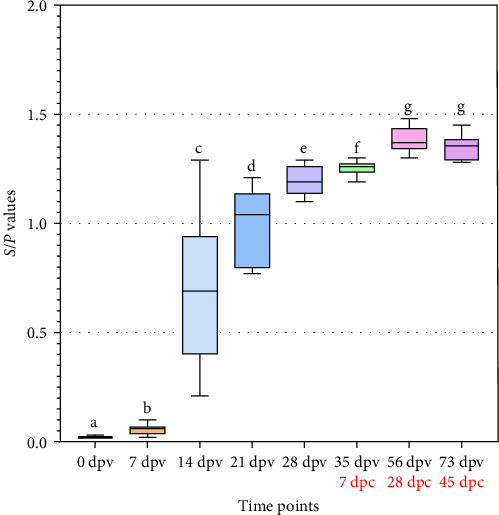
*S*/*P* values indicate anti-ASFV antibody levels post-vaccination and post-challenge. Different letters indicate significant differences between groups (*p* < 0.05), while the same letter indicates no significant differences between groups (*p* > 0.05).

The results of testing serum samples from vaccinated pigs and control pigs at 7, 14, 21, and 28 dpv demonstrated a gradual increase in the number of vaccinated pigs testing positive for anti-ASFV antibodies over time. The percentage increased from 0% at day 7 to 80% (8/10) at day 14, and reached 100% at days 21 and 28 post-vaccination. This percentage was maintained at 7, 28, and 45 dpc (Figure 8). Concurrently, the *S*/*P* values also increased gradually over time, starting from 0.70 ± 0.34 at 14 dpv, rising to 0.98 ± 0.17 at 21 dpv, and reaching 1.19 ± 0.07 at 28 dpv. After challenge, the S/*P* values increased to 1.26 ± 0.04 at 7 dpc and up to 1.35 ± 0.06 at 45 dpc. The *S*/P values of pigs measured at various time points were significant different (*p* < 0.05), except for no significant difference between 56 and 73 dpv, corresponding to 28 and 45 dpc (*p* > 0.05; Figure 9). Meanwhile, all control pigs consistently tested negative for anti-ASFV antibodies throughout the experiment (Figure 8).

### 3.4. Duration of Protective Immunity

A study on the duration of protective immunity involved 46 healthy 4-week-old pigs divided into two groups. The vaccinated group consisted of 28 pigs (ear-tag: D1–D28) receiving a vaccine dose, while the control group had 18 pigs (ear-tag: D29–D46). Serum samples were collected at 14, 28, 90, 120, 150, and 180 dpv to test for anti-ASFV antibodies. At 14, 90, 120, 150, and 180 dpv, five vaccinated pigs and three control pigs were randomly selected for challenge with virulent ASFV. Challenged pigs were monitored daily for 21 days for clinical signs. In case of death or reaching the humane endpoint, a necropsy was performed to determine the cause of death. Pigs were considered protected if they survived the challenge without showing typical clinical signs of acute or chronic ASF.

**Figure 10:**
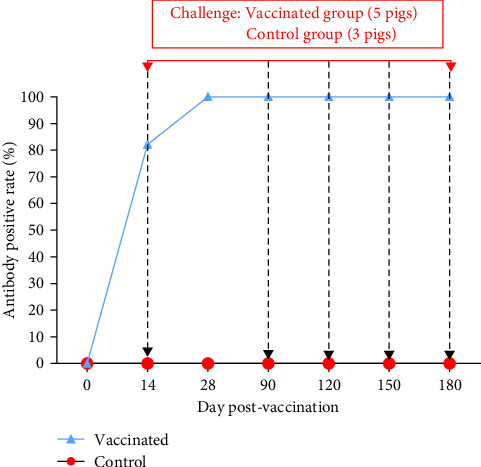
Positive rate for anti-ASFV antibodies at various time points post-vaccination.

**Figure 11:**
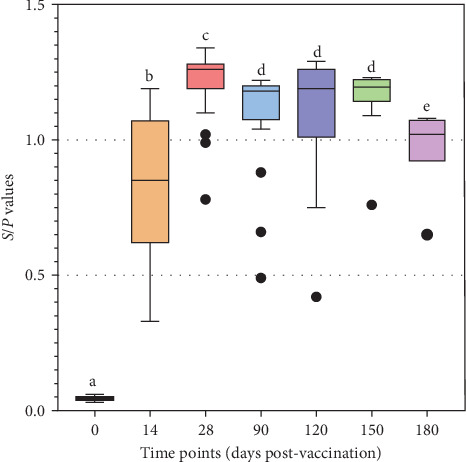
*S*/*P* values indicate anti-ASFV antibody levels at various time points. Different letters indicate significant differences between groups (*p* < 0.05), while the same letter indicates no significant differences between groups (*p* > 0.05). Dots indicate outliers.

Similar to the efficacy experiment, the number of vaccinated pigs testing positive for anti-ASFV antibodies increased gradually over time. Initially undetected on day 7, the percentage rose to 82.14% at day 14 and reached 100% by day 28 post-vaccination, maintaining this rate until 180 dpv (Figure 10). Statistical analysis of *S*/*P* values identified outliers in one to three samples at 28, 90, 120, 150, and 180 dpv, likely due to individual differences, which were excluded from the analysis. The results indicated that *S*/*P* values increased from 0.82 ± 0.26 at day 14 to a peak of 1.25 ± 0.05 at day 28 post-vaccination (*p* < 0.05). The *S*/*P* values remained stable at 1.17 ± 0.05 at 90 dpv, 1.15 ± 0.15 at 120 dpv, and 1.19 ± 0.04 at 150 dpv, with no significant differences observed among these time points (*p* values > 0.05). Subsequently, the *S*/*P* values decreased to 1.02 ± 0.07 at 180 dpv (*p* < 0.05; Figure 11).

**Figure 12:**
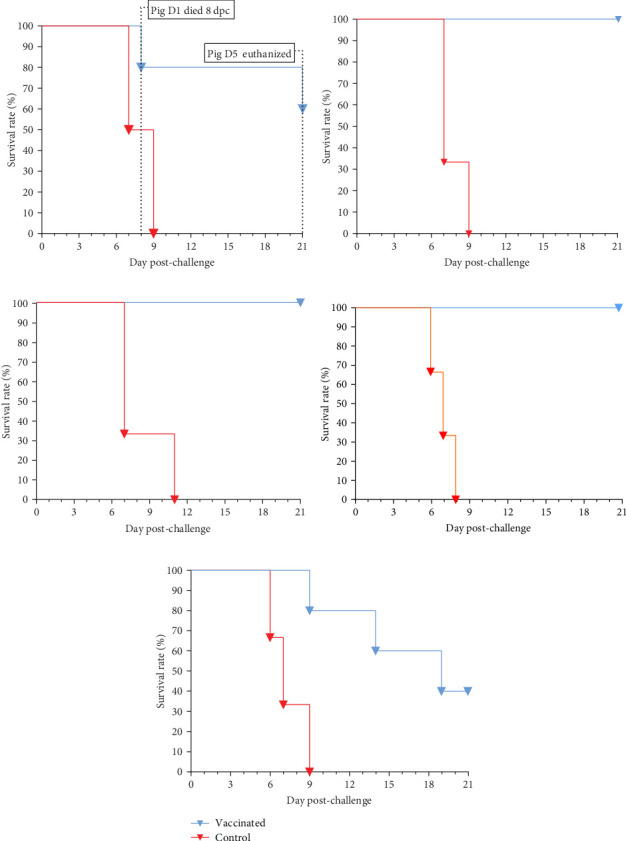
Survival rates of pigs challenged at various time points post-vaccination. (a) Challenge at 14 dpv, (b) challenge at 90 dpv, (c) challenge at 120 dpv; (d) challenge at 150 dpv; and (e) challenge at 180 dpv.

The results of challenge experiments conducted at 14, 90, 120, 150, and 180 dpv showed that all control pigs exhibited typical signs of ASF and died between 6–11 dpc (Figure 12). Among vaccinated pigs, 100% of pigs survived when challenged at 90, 120, and 150 dpv, while the survival rates for groups challenged at 14 and 180 dpv were 60% (3/5) and 40% (2/5), respectively (Figure 12).

In the group challenged at 14 dpv, one pig that tested negative for anti-ASFV antibodies exhibited typical clinical signs of ASF and died at 8 dpc, showing typical ASF lesions and a high ASFV load in the spleen sample (Ct = 18.9). Another pig (D5) demonstrated seroconversion but developed clinical signs of fever, poor appetite, and arthritis from day 11 to day 21 post-challenge. This pig was humanely sacrificed for pathological examination and ASFV testing at the end of the 21-day observation period post-challenge. The necropsy examination also revealed typical ASF lesions and the presence of the virus in the blood (Ct = 28.84) and spleen (Ct = 22.17).

In the group challenged at 180 dpv, five vaccinated pigs still tested positive for anti-ASFV antibodies. Among them, three pigs exhibited clinical signs of ASF and died between day 9 and 18 post-challenge. Postmortem examination confirmed the presence of typical ASF lesions.

## 4. Discussion

In this paper, we present the safety and efficacy profiles of the AVAC ASF LIVE vaccine designed to prevent ASF in pigs. The vaccine demonstrated its safety in 4-week-old pigs as the vaccinated pigs remained healthy with no abnormal signs even when administered a dose 100 times higher than the recommended protective dose. After receiving a single protective dose, the percentage of vaccinated pigs testing positive for anti-ASFV antibodies increased, reaching 100% by day 21 post-vaccination and maintaining that level until 180 dpv. Only a few pigs developed low viremia on certain dpv and dpc, but became negative from 28 dpv and dpc. They did not shed viruses in oral–nasal swabs or rectal swabs after 7 dpc. Additionally, a single dose of the vaccine effectively provided complete protection for 60% of vaccinated pigs against a highly virulent field virus of genotype II when challenged at 14 dpv. The survival rate reached 100% when challenged at 28, 90, 120, and 150 dpv, indicating durable protective immunity that effectively prevents genotype II ASFV infection in vaccinated pigs until slaughter.

So far, only two commercial vaccines for ASF have been licensed for use in Vietnam [11, 12]. Both vaccines are derived from the highly virulent pandemic ASFV strain Georgia (ASFV-G). The NAVET-ASFVAC vaccine uses a modified virus strain, ASFV-G-∆I177L, created by deleting the I177L gene from the original virus [24–27]. The AVAC ASF LIVE vaccine (ASFV-G-ΔMGF-DMAC) was developed by further attenuating the ASFV-G-ΔMGF strain, which involved deleting six genes from the multigene family 360 (MGF360) and MGF505 of the parental strain (ASFV-G) [13], in DMAC cells. Both attenuated strains, ASFV-G-∆I177L and ASFV-G-ΔMGF, have been proven safe for pigs and effective in protecting them from challenges with the parental virulent virus [13, 27–31]. The ASFV-G-ΔMGF strain did not show significant virulent reversion after five consecutive passages through pigs [28]. The results of this study suggest that the AVAC ASF LIVE vaccine demonstrates similar clinical safety and protective efficacy to the original virus strain (ASFV-G-ΔMGF) [13, 29] and ASFV-G-∆I177L strain [24–27].

However, significant differences were observed in viremia and viral shedding in secretions among the vaccines. All pigs injected with ASFV-G-∆I177L strain exhibited viremia from 7 to 21 dpv and transmitted the vaccine virus to contact pigs [27]. Similarly, 80%–100% of animals inoculated with the ASFV-G-ΔMGF strain showed prolonged viremia until 28 dpv. They were protected against clinical disease, but 30% to 40% of them carried the challenge virus (ASFV-G) in their blood samples at 28 dpc [13]. In contrast, only 50% of pigs that received ASFV-G-ΔMGF-DMAC had low viremia at 7 dpv, decreasing to 30% at 14 dpv, and all pigs tested negative by 21 dpv. None of them shed the vaccine virus in secretions post-vaccination. After the challenge, the percentage of pigs with viremia peaked at 60% on day 7, then dropped to 10% on day 28, and became negative by day 35. Only 10%–40% of vaccinated pigs shed the virus in nasal swabs at 4, 7, and 10 dpc with low levels of viral DNA. Additionally, upon necropsy at 45 dpc, only two out of 10 (20%) vaccinated pigs were found to harbor low challenge virus DNA in internal organs. This suggests that, compared to the other vaccine candidates, the AVAC ASF LIVE vaccine poses a lower risk of vaccinated animals shedding the vaccine virus into the environment. This reduces the risk of recombination with field viruses and helps avoid further complications in the already complex ASF situation. It should be noted that follow-up periods after vaccination and challenge vary between studies. Most studies typically conducted follow-up periods of 21–45 dpv and dpc [13, 25, 28]. Our study also applied a follow-up of 45 dpv and dpc, consistent with the draft guidelines established by the World Organization for Animal Health, recommending a follow-up period of 45–60 days after vaccination and challenge [14]. However, some studies on naturally attenuated strains NH/P68 and OURT88/3 have monitored protective immunity for up to 120–134 days after vaccination [30, 31], with follow-up to 85 dpc [30]. Research has shown that pigs inoculated with attenuated ASFV strains or those that survive virulent infection may experience a recurrence of clinical signs 50–60 days post-inoculation [30, 32]. In addition, virulent ASFV strains can remain undetected in tissues of clinically recovered animals, but viral replication can resume after this period. Therefore, a longer follow-up period after vaccination and challenge is necessary to evaluate ASFV persistence and delayed adverse effects following vaccination.

In this study, we used MGF360-12L and β-GUS genes as markers to differentiate between infected and vaccinated animals. However, six samples tested positive for B646L with a Ct value above 36.0 but tested negative for both MGF360-12L and β-GUS genes. This suggests that these markers may have lower sensitivity compared to B646L for detecting ASFV. Further extensive trials with a larger sample size would be necessary to assess the sensitivity and reliability of MGF360-12L and β-GUS genes as DIVA markers. Moreover, since modified live vaccines and attenuated ASFV strains often lead to low or undetectable viremia, qPCR-based DIVA strategies may be ineffective, highlighting the importance of serological DIVA as a more reliable and faster approach. Future studies should also include virus isolation assays to confirm if ASFV remains infectious in vaccinated pigs.

The duration of protective immunity is a critical factor in assessing vaccine effectiveness, as it varies among different vaccines. Some vaccines provide lifelong protection with a single dose, while others require booster shots [33]. Therefore, determining the duration of immunity for each vaccine is essential to establish an appropriate vaccination schedule. However, most ASF vaccine candidates are still in laboratory clinical trials and have not been commercialized [9], leading to limited research on the duration of protective immunity induced by ASF vaccines in pigs [34]. Most vaccine candidates were assessed in experimental settings with challenges occurring a few weeks after vaccination [9]. The protective efficacy of ASFV-G-∆I177L strain, used in a commercial vaccine, was also evaluated by challenging pigs at 14, 21, and 28 dpv [25]. These time points of challenge in a short period are insufficient to determine the duration of immunity.

Only one study monitored the immune response of experimental pigs over a relatively long period of 130 dpv with two attenuated strains: the naturally attenuated OURT88/3 strain and the BeninΔMGF strain. However, the inoculated pigs were challenged at only one time point of 130 dpv, and they were not protected against the challenge dose [34]. This experimental design, with the data collected, was insufficient to determine the duration of protective immunity. Thus, the present study is the first to ascertain the duration of immunity for ASF vaccines by monitoring and challenging pigs at various time points in a 180-day period. All vaccinated pigs survived the challenges at 28, 90, 120, and 150 dpv, with a 40% survival rate at 180 dpv, suggesting protective immunity lasting for at least 150 days (5 months), which aligns with the typical marketing time for finishing pigs. At 180 dpv, while antibody positivity remained at 100%, the S/P index decreased to less than 1.0, and the protective rate against challenges was low. This decline may be attributed to waning cellular immunity, which plays a crucial role in ASF immunity [30, 34]. The protective immunity response to ASFV infection involves both humoral and cell-mediated mechanisms, although the exact mechanism is not fully understood [10]. Antibodies transferred through colostrum or serum from convalescent pigs have shown protective activity against ASFV. While ASFV cannot be completely neutralized in vitro, antibodies from convalescent or protected pigs can kill ASFV-infected cells through complement-mediated cytotoxicity and antibody-dependent cellular cytotoxicity. Other studies have shown that CD8+ lymphocytes play a crucial role in protective immunity [30]. Pigs immunized with CD8+ lymphocyte depletion were not fully protected from virulent strain challenge, suggesting that anti-ASFV antibodies alone may not be sufficient for protection. Conversely, viremic pigs depleted of CD8+ lymphocytes still survived the challenge, indicating that other factors, such as antibodies, may also contribute to protective immunity against ASFV. Therefore, evaluating both cellular and humoral immune responses is essential to understand the protective immunity induced by ASF-G-ΔMGF-DMAC.

It should be noted that a new highly pathogenic recombinant strain (rASFV GI/GII) has emerged, complicating the ASF situation. Additionally, two commercial vaccines in Vietnam do not provide protection for pigs against this recombinant strain [12]. This highlights the necessity for new vaccine strategies to effectively address emerging recombinant variants. However, the recombinant strain has only been detected in limited areas in China [35], Vietnam [36], and recently in Russia [37]. Therefore, while a vaccine against the recombinant strain or both genotype II and recombinant strains has not been developed, the administration of these commercially available vaccines can protect pig herds against at least genotype II ASFV, which is currently predominant in Vietnam and other countries.

Outside of Vietnam, the AVAC ASF LIVE vaccine was tested in the Philippines, where the trial results showed a 100% production of anti-ASFV antibodies among vaccinated 4- to 10-week-old pigs, with no clinical signs of ASF and no evidence of viral shedding. Based on these positive results, the Food and Drug Administration, Ministry of Health approved the certificate of product registration for restricted use by the Department of Agriculture-Bureau of Animal Industry, for 2 years beginning July 2024. The Philippine government is currently procuring ASF vaccines to initiate widespread vaccination [38–40].

While the battle against ASF is recognized as a long-term effort, the initial successes in commercializing ASF vaccines in Vietnam provide optimism that the ASF outbreak will be brought under control. With the development and approval of more vaccines that are effective against virulent field strains on a global scale, we anticipate continued progress in the fight against this economically devastating disease.

## 5. Conclusions

The AVAC ASF LIVE vaccine, produced from the live attenuated ASFV strain ASFV-G-ΔMGF-DMAC, has proven to be safe and highly effective for pigs. When young pigs were administered a dose 100 times higher than the protective dose at 4 weeks old, they showed no adverse effects and behaved normally. Following a single dose, vaccinated pigs developed strong immunity by day 14 and remained protected against the highly virulent genotype II virus for up to 150 dpv. Additionally, vaccinated pigs did not shed the vaccine virus in secretions and minimized the spread of challenge viruses. Thus, the AVAC ASF LIVE vaccine provides a safe and effective solution for preventing genotype II ASFV infection.

## Figures and Tables

**Table 1:** Experimental design for safety test of the vaccine in young animals.

Activities	Immunized pigs	Control pigs
Ear tag	S1–S10	S11–S20

Vaccination	10^6^ HAD_50_ ASFV-G-ΔMGF-DMAC per pig (equivalent to 100 doses)	None

Follow-up for 45 day period	+ Rectal temperature measured daily, clinical signs and clinical scores were recorded. + Pigs that reach a clinical score from 10 points were humanely euthanized for gross pathology examination. + At the end of the experiments, all pigs were euthanized and subjected to postmortem examination.	

**Table 2:** Experimental design for efficacy test of the vaccine.

Activities	Immunized pigs	Control pigs	Follow-up
Ear tag	E1–E10	E11–E15	—

Vaccination	10^4^ HAD_50_ of ASFV-G-ΔMGF-DMAC (a dose) per pig	None	+ Monitored daily for clinical signs and rectal temperatures for a 28-day period. + Blood samples and oral swabs were collected at 7, 14, 21, and 28 dpv to test for ASFV DNA and anti-ASFV antibodies.

Challenge at 28 dpv	10^3^ HAD_50_ of Hv-Avac01 per pig		+ Follow-up for 45 days for clinical signs and rectal temperature. + Blood, serum, oral–nasal, and rectal swab samples were collected at 4, 7, 10, 14, 21, 28, 35, and 45 dpc to test for ASFV DNA and anti-ASFV antibodies. + Necropsy of dead pigs to determine the cause of death. + After 45 days, all surviving experimental pigs were humanely euthanized and necropsied to examine gross pathology.

**Table 3:** Experimental design for duration of protective immunity.

Time (dpv)	Activities	Immuned pigs	Control pigs	Follow-up
	Ear tag	D1–D28	D29–D46	
D0	Vaccination	One vaccine dose per pig	None	+ Antibody tests for all live pigs at these time points, as well as at 28 days post-vaccination, before challenge. + Follow-up for 21 days post-challenge. + Viremia tests at 4, 7, 14, and 21 days post-challenge. + Survival rates post-challenge recorded.
D14	Challenge	D1–D5	C29–C31	
D90	Challenge	D6–D10	C32–C34	
D120	Challenge	D11–D15	C35–C37	
D150	Challenge	D16–D20	C38–C40	
D180	Challenge	D21–D25	C41–C43	

*Note:* The challenge at 28 days post-vaccination was already conducted in the efficacy test and was not repeated to reduce the number of animals. Each group has three extra pigs.

**Table 4:** Genetic changes of ASFV-G-ΔMGF-DMAC compared to the ASFV-G-ΔMGF strain.

Nucleotide position	Gene	Mutation	Amino acid change
13257	MGF 110–10 L	Deletion (C)	Frame shift
13258	MGF110-14 L	Deletion (C)	Frame shift
21601	MGF 300-2R	Insertion (A)	Frame shift
75596	M448R	T → C mutation	Tyr → His
76341	Hypothetical protein coding gene	Insertion (T)	Nonsense mutation
119624 119625	CP204L	Insertion (CC)	Stop codon at amino acid 186
133245	D129L	Insertion (T)	Silent mutation
156121	QP383R	Insertion (A)	Frame shift
164569	I267L	Insertion (T)	Stop codon at amino acid 267
172330	MGF 360-16R	Insertion (A)	Frame shift
51 mutation sites	Nonprotein-coding regions	51 single-nucleotide polymorphism (SNP) sites	—

**Table 5:** Detection of ASFV DNA in the internal organs of pigs 45 days post-challenge.

Pig's ear tag	Target gene	Spleen	Kidney	Lymph node	Lung
E1	B646L	—	—	—	—

E2	B646L	36.33	36.28	36.51	—
	MGF360 12 L	—	—	—	—
	β-GUS	—	—	—	—

E3	B646L	—	—	—	—

E4	B646L	36.8	—	34.41	36.46
	MGF360 12 L	—	—	33.75	—
	β-GUS	—	—	—	—

E5	B646L	35.58	—	34.03	—
	MGF360 12 L	—	—	—	—
	β-GUS	—	—	35.18	—

E6	B646L	—	—	—	—
	MGF360 12 L	—	—	—	—
	β-GUS	—	—	—	—

E7	B646L	34.72	35.52	32.00	33.59
	MGF360 12 L	36.93	35.74	35.20	32.11
	β-GUS	—	—	—	—

E8	B646L	—	—	—	—

E9	B646L	—	—	—	—

E10	B646L	—	—	—	—

## Data Availability

The data that support the findings of this study are available from the corresponding author upon reasonable request.
